# Cooperation between Adh and OR5M11 contributes to the lung pathogenicity of *Actinobacillus pleuropneumoniae* by facilitating the NLRP3-mediated inflammatory response

**DOI:** 10.1186/s13567-026-01845-4

**Published:** 2026-10-03

**Authors:** Bo Wen, Xiaoli Yin, Xinhe Zhang, Chunling Zhu, Xiaofeng Li, Yilin Bai, Huarun Sun, Zhiyi Liu, Shuwen Ji, Ruibiao Wang, Chenru Yu, Jiaqi Wang, Longfei Zhang, Jiyuan Shen, Jianhe Hu, Yueyu Bai, Huihui Zhang, Ke Ding, Lei Wang

**Affiliations:** 1 Henan Institute of Science and Technology, the Pingyuan Laboratory, Xinxiang, China; 2 Ministry of Education Key Laboratory for Animal Pathogens and Biosafety, Zhengzhou, China; 3 Laboratory of Functional Microbiology and Animal Health, Henan University of Science and Technology, Luoyang, China; 4 School of Agricultural Sciences, Zhengzhou University, Zhengzhou, Henan China

**Keywords:** *Actinobacillus pleuropneumoniae*, Adh, OR5M11, NLRP3, hyperinflammation, lung injury

## Abstract

*Actinobacillus pleuropneumoniae* (*A. pleuropneumoniae*) is the etiologic agent of porcine pleuropneumonia, a severe respiratory disease characterized by hemorrhagic, necrotizing lung lesions that cause significant economic losses on the global swine industry. However, the molecular mechanisms underlying the lung pathogenicity of *A. pleuropneumoniae* remain unclear. Here we demonstrate that *A. pleuropneumoniae* infection induces lung injury through activating the NLRP3-mediated inflammatory response mediated by the YadA-like head region (Adh) of Apa1, a bacterial adhesin that mediates attachment to host cells. Importantly, we show that Adh triggers the NLRP3-mediated inflammatory response by engaging olfactory receptor family 5 subfamily M member 11 (OR5M11), a host receptor previously shown to interact with Adh. Furthermore, OR5M11 contributes to the Adh-activated inflammatory response by promoting mitochondrial DNA release. Taken together, our results provide new insights into the pathogenesis of *A. pleuropneumoniae,* particularly regarding NLRP3-mediated inflammatory pathology*.*

**Supplementary Information:**

The online version contains supplementary material available at https://doi.org/10.1186/s13567-026-01845-4.

## Introduction

Porcine pleuropneumonia is a highly contagious fatal disease which causes substantial economic losses to the pig industry worldwide by affecting swine of all ages [1]. The causative agent of porcine pleuropneumonia, *A. pleuropneumoniae*, belongs to the family *Pasteurellaceae*, and has been divided into 19 serotypes [2]. A lack of cross-protection between the main serovars results in slow progress in the development of vaccines, making the infection very difficult to control [3]. *A. pleuropneumoniae* is a respiratory pathogen and usually induces dyspnea, cough, high fever, and peripheral cyanosis of extremities. *A. pleuropneumoniae* exhibits typical lung pathogenicity characterized by severe congestion and fibrosis in the lungs, with necrotic lesions commonly observed, accompanied by considerable inflammatory cell infiltration and severe structural damage of the alveoli [4, 5]. The excessive inflammatory immune response triggered by *A. pleuropneumoniae* in the lungs is a key cause of the serious lung injury [1, 6–9]. However, the molecular mechanisms that underpin the lung pathogenicity of *A. pleuropneumoniae* remain unclear.

Trimeric autotransporter adhesins (TAAs) are bacterial adhesion structures that participate in adhesion, biofilm formation, autoagglutination, and early bacterial pathogenic processes [10, 11]. TAAs have been observed on many bacteria, such as YadA on the surface of *Yersinia enterocolitica*, NadA on *Neisseria meningitidis*, UpaG on *uropathogenic Escherichia coli*, BadA on *Bartonella henselae*, BtaE on *Brucella*, and HadA on the cell surface of *Haemophilus influenzae* [12–17]. *A. pleuropneumoniae* also possesses two TAA proteins, Apa1 and Apa2, which mediate bacterial autoagglutination and adherence to host cells [18, 19]. Amino acids 68–612 located in the head region of Apa1, named Adh, have been demonstrated to constitute the main functional domain, playing an important role in *A. pleuropneumoniae* adhesion to host cells, autoagglutination, and biofilm formation [20]. Furthermore, Adh contributes to the early-phase pathogenicity of *A. pleuropneumoniae*, particularly lung pathogenicity, through excessive inflammatory immune responses [20, 21]. Interestingly, olfactory receptor family 5 subfamily M member 11 (OR5M11) was identified to interact with Adh, which mediates Adh-induced release of inflammatory cytokines [20].

Inflammasomes are macromolecular multimeric protein complexes composed of intracellular pattern recognition receptors (PRRs), the adaptor protein apoptosis-associated speck-like protein containing a CARD (ASC), and the effector pro-Caspase−1 (pro-CASP-1) [22]. The nucleotide-binding domain and leucine-rich repeat containing receptor (NLR) family PYRIN domain containing 3 (NLRP3) inflammasome is one of the best-characterized inflammasomes. Upon activation, the NLRP3 inflammasome serves as a platform for caspase−1 activation, resulting in caspase−1-dependent proteolytic maturation of pro-interleukin-1β (IL-1β) [23]. As an essential innate immune defense mechanism, inflammasomes are usually activated during cellular stress and infections [24, 25]. However, aberrant activation of the NLRP3 inflammasome triggers excessive release of pro-inflammatory cytokines, including IL-1β, IL-18, and IL-6, potentially leading to a “cytokine storm”, skewing the host response toward pathological inflammatory events [26–28]. Although many studies highlight that excessive activation of the NLRP3 inflammasome results in hyperinflammation and severe tissue damage, whether the inflammatory lung injury induced by *A. pleuropneumoniae* is mediated by aberrant activation of the NLRP3 inflammasome has not been determined.

In this study, we investigated the activation of the NLRP3 inflammasome and its role in *A. pleuropneumoniae* infection-induced inflammatory lung injury in vitro and in vivo, and identified the mechanism underlying the pathogenesis of *A. pleuropneumoniae* infection.

## Materials and methods

### Ethics statement

All animal studies were conducted according to the experimental practices and standards of the Animal Welfare and Research Ethics Committee at Zhengzhou University. The study was also approved by the Animal Center of Zhengzhou University (IACUC license number: no. 41003100024648). All the animals were humanely handled.

### Bacterial strains and cells

*Actinobacillus pleuropneumoniae* serotype 5 strain L20 (5b WT) was obtained from the Shanghai Entry-Exit Inspection and Quarantine Bureau. The trimeric autotransporter adhesins-deletion strain (5b *ΔAdh*) was constructed [21] and stored by our laboratory. Primary PAMs were collected from the BALF of healthy weaned piglets, which were purchased from a pig-breeding farm. The PAM cell line (ATCC CRL-2845) was kindly provided by Professor Feng (China Agricultural University). These cells were grown in RPMI-1640 (Gibco, Carlsbad, USA) supplemented with 10% foetal bovine serum (FBS), 2 mM L-glutamine, 100 U/mL penicillin, and 100 μg/mL streptomycin.

### Experimental design and groupings

**Figure 1:**
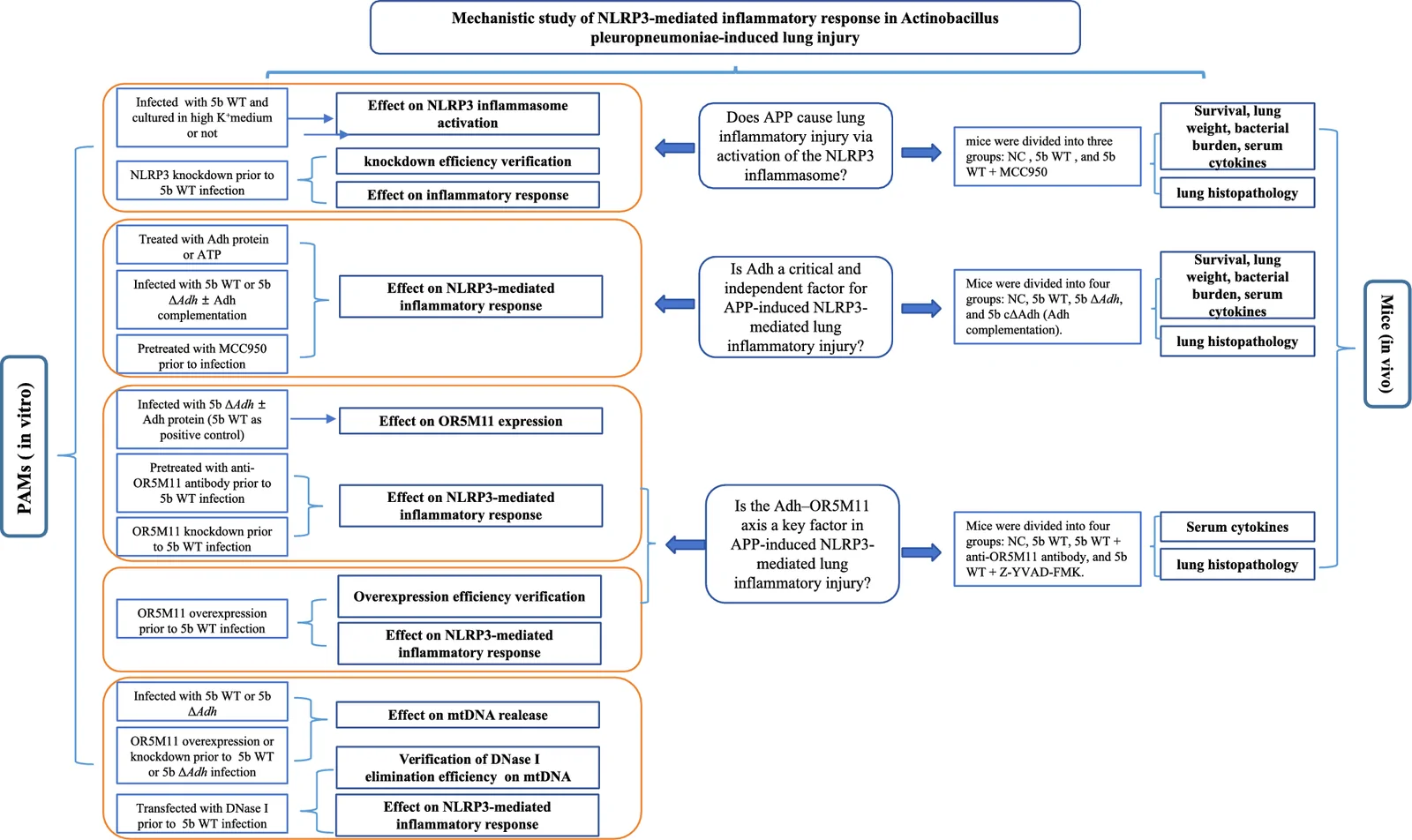
**A schematic flow chart illustrating the overall experimental design.**

All in vitro experiments were performed with PAMs unless otherwise specified. For infection experiments, PAMs were infected with *A. pleuropneumoniae* 5b WT or 5b Δ*Adh* at an MOI of 10 for 12 h. Untreated cells served as the negative control (NT or NC). A schematic flow chart illustrates the overall experimental design (Figure 1).

In vitro bacterial infection and cell culture models—detailing PAM culture conditions and standardized infection protocols (5b WT and 5b ΔAdh, MOI, duration).

Pharmacological and antibody treatment—consolidating all drug and antibody intervention regimens (MCC950, Z-YVAD-FMK, anti-OR5M11 antibody, DNase I), including dosages, pretreatment times, and rationale.

Genetic manipulation—describing siRNA transfection (si-NLRP3, si-OR5M11) and plasmid overexpression (pCMV-OR5M11) procedures, concentration optimization, and validation.

Protein and nucleic acid detection—presenting western blotting, qRT-PCR, and ELISA methodologies as unified analytical platforms.

Mitochondrial DNA release assays—detailing cytosolic fraction isolation and mtDNA quantification (cyt b, COX I, D-loop, ND1).

In vivo infection model and histopathological analysis—describing mouse grouping, infection parameters, treatments, and tissue processing.

### Antibodies and reagents

Primary antibody against caspase−1 (24232), Cle-caspase−1 (24232), AIM2 (53491), IL-1β (12426), Cle-IL-1β (12426), and ASC (67824) were purchased from cell signalling technology. Primary antibody against GSDMD (PU224937) and Cle-GSDMD (PU224937) were purchased from abmart. Primary antibody against OR5M11 (DF5110) was purchased from affinity. Primary antibody against β-actin (HC201) was purchased from Transgen. HRP-conjugated goat anti-mouse IgG (A9917), and HRP-conjugated goat anti-rabbit IgG (A0545) were from Sigma. Cell Mitochondrial isolation kit (C3601) was purchased from Beyotime. DNA Isolation kit (69504) was purchased from QIAGEN. DNase I (E127–01B) was purchased from Novoprotein. MCC950 (HY-12815) and Z-YVAD-FMK (HY-P1009) were purchased from MedChemExpress.

### Western blotting

The harvested cells were treated with RIPA lysis buffer containing phenylmethyl sulfonylfluoride (PMSF) for generating cell lysates. Protein samples were produced by adding 5× sodium dodecyl sulfate (SDS)-polyacrylamide gel electrophoresis (PAGE) sample buffer to lysed cells. The samples were boiled for 10 min, separated by 12% sodium dodecyl sulfate–polyacrylamide gel electrophoresis (SDS-PAGE), and then transferred onto 0.22 µm polyvinylidene difluoride membranes (Millipore, Billerica, MA, USA). The membranes were blocked with 5–10% nonfat milk in TBST for 2 h and then probed overnight at 4 °C with primary antibodies. After washing, the membranes were reacted with HRP-conjugated secondary antibodies at room temperature for 1 h. At last, the bound antibodies were detected with enhanced chemiluminescence immunoblotting detection reagents (Millipore). Images were obtained with a CanoScan LiDE 100 scanner (Canon).

### RNA isolation and Realtime quantitative RT-PCR

qRT-PCR was used to verify the cytokine, apoptotic signaling pathway-associated protein. The primer sequences are listed in Additional file 1. RNA extraction and reverse transcription was performed with the TaKaRa RT reagent kit with gDNA Eraser (Perfect Real Time, lot no. RR047A) according to the manufacturer’s instructions.

### Transient transfections and chemical treatment

All plasmids and siRNA (Ribo, Guangzhou, China) transfections were performed using TurboFect Transfection Reagent (Thermo Fisher Scientific, R0531) according to the manufacturer’s protocol. MCC950 (MCE, HY-12815) was used to pre-treat cells. The treatments were applied 2 h before bacterial strains infection and maintained until the end of the experiments.

### Hematoxylin and Eosin (HE) staining

The fixed lung samples were dehydrated, embedded in paraffin, and serial sectioned (4 mm). Three sagittal sections from each lung, and six sections per animal, were stained with hematoxylin–eosin and Masson’s trichrome. The severities of histopathological changes and pulmonary fibrosis of the lungs were assessed and scored. All the sections were numbered randomly and interpreted by three experimenters blinded to the experimental conditions. Histopathological changes were observed and scored under an Olympus microscope (Olympus Optical Co., Ltd.).

### Determination of mtDNA copies in cytosolic extracts

The cytoplasmic fraction of PAMs is isolated using the Cell Mitochondrial Isolation Kit (C3601, Beyotime Biotechnology) according to the instructions. Briefly, 10^7^ cells were incubated in 0.5 mL of mitochondrial lysis buffer for 10 min and then homogenized 30 times with Dounce homogenizer. The supernatants are collected and determined the cytoplasmic mtDNA concentration using qRT-PCR.

### Statistical analysis

All experiments were repeated at least three times. The data are represented as the mean ± SD or typical photographs and are representative of three independent experiments. For data with two groups, unpaired Student’s *t*-tests were used under the assumption of normality. Data with more than two groups were analyzed by analysis of variance (ANOVA) under assumption of normality. Values of *P* < 0.05 were considered statistically significant. All quantitative data were analyzed using GraphPad Prism 8 (GraphPad Software, inc. USA).

## Results

### *Actinobacillus pleuropneumoniae* induces the NLRP3-mediated inflammatory response

**Figure 2:**
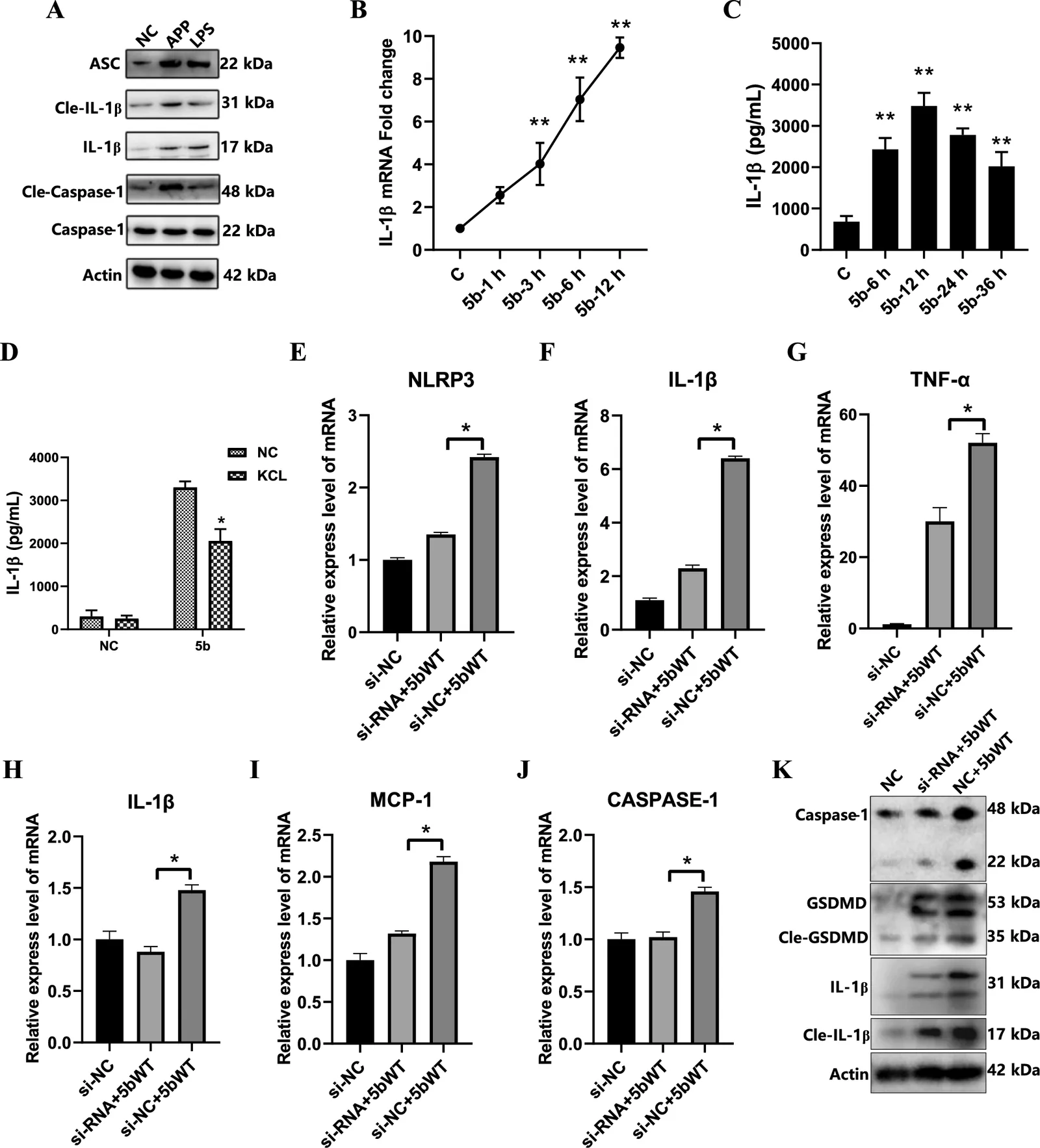
***Actinobacillus pleuropneumoniae* infection activates NLRP3-mediated inflammatory response**. **A** PAMs were infected with 5b WT at 10 MOI. 12 h later, the expression of NLRP3 inflammasome related proteins were detected by western blot. **B** and **C** PAMs were infected with 5b WT at 10 MOI, the mRNA levels (**B**) and protein level (**C**) of IL-1β were tested by qRT-PCR and ELISA respectively, at different time points. **D** ELISA assay for IL-1β in PAMs cultured in high-content K^+^ cell culture system was measured. **E**–**J** The mRNA levels of cytokines in NLRP3 knockdown PAMs. PAMs were transfected with nonspecific control siRNA (NC) or siRNA against NLRP3 (si-RNA) for 12 h and then infected with 5b WT at an MOI of 10 for 12 h. Then, the cells were subjected to qRT-PCR analysis. **K** PAMs were transfected with NC or si-RNA for 12 h and then infected with 5b WT at an MOI of 10 for 12 h. Then, the cells were subjected to western blot analysis. The levels of NLRP3 inflammasome related proteins in NLRP3 knockdown PAMs. Error bars indicate the mean (± SD) of three repeats. The data are representative of results from three independent experiments with similar results. *P < 0.05; **P < 0.05. NS, not significant.

The NLRP3-mediated inflammatory response (mainly the activation of the NLRP3 inflammasome) has been shown to be involved in inflammatory lung injury [29–31]. However, the relationship between the lung pathogenicity of *A. pleuropneumoniae* and the NLRP3-mediated inflammatory response remains unknown. First, we evaluated the expression of NLRP3 inflammasome-related molecules in *A. pleuropneumoniae* (serotype 5 strain L20, 5b WT)-infected porcine alveolar macrophages (PAMs). We detected elevated levels of ASC, mature caspase−1 (p10), and IL-1β (p17) by western blot in 5b WT-infected PAMs at 12 hpi, and in the positive group (LPS-treated), compared with the mock-infected group (NC) (Figure 2A and Additional file 2). Consistent with these findings, the mRNA levels of IL-1β in 5b WT-infected PAMs were upregulated in a time-dependent manner relative to the control group (Figure 2B). ELISA analysis showed that IL-1β protein levels in the supernatant of 5b WT-infected PAMs began to increase dramatically at 6 hours post-infection (hpi), peaking at 12 hpi (Figure 2C). It has been reported that K^+^ efflux is a common trigger of NLRP3 inflammasome activation by bacterial toxins [32]. Here, we found that inhibition of K⁺efflux by culturing PAMs in high-K^+^ medium decreased IL-1β levels in the supernatant of 5b WT-infected PAMs (Figure 2D). Collectively, these results indicate that 5b WT activates the NLRP3 inflammasome in vitro.

To examine the role of the NLRP3 inflammasome in 5b WT-induced inflammatory responses, we used RNA interference to suppress NLRP3 expression in PAMs. When NLRP3 was knocked down (Figure 2E), pro-inflammatory cytokines (IL-18, IL-1β, TNF-α, and MCP-1) were downregulated (Figure 2F–J), and the cleavage of caspase−1, GSDMD, and IL-1β was attenuated compared with the negative control (NC) group for RNA interference during 5b WT infection (Figure 2K and Additional file 2).

### *Actinobacillus pleuropneumoniae* causes lung injury via NLRP3-mediated inflammatory response

**Figure 3:**
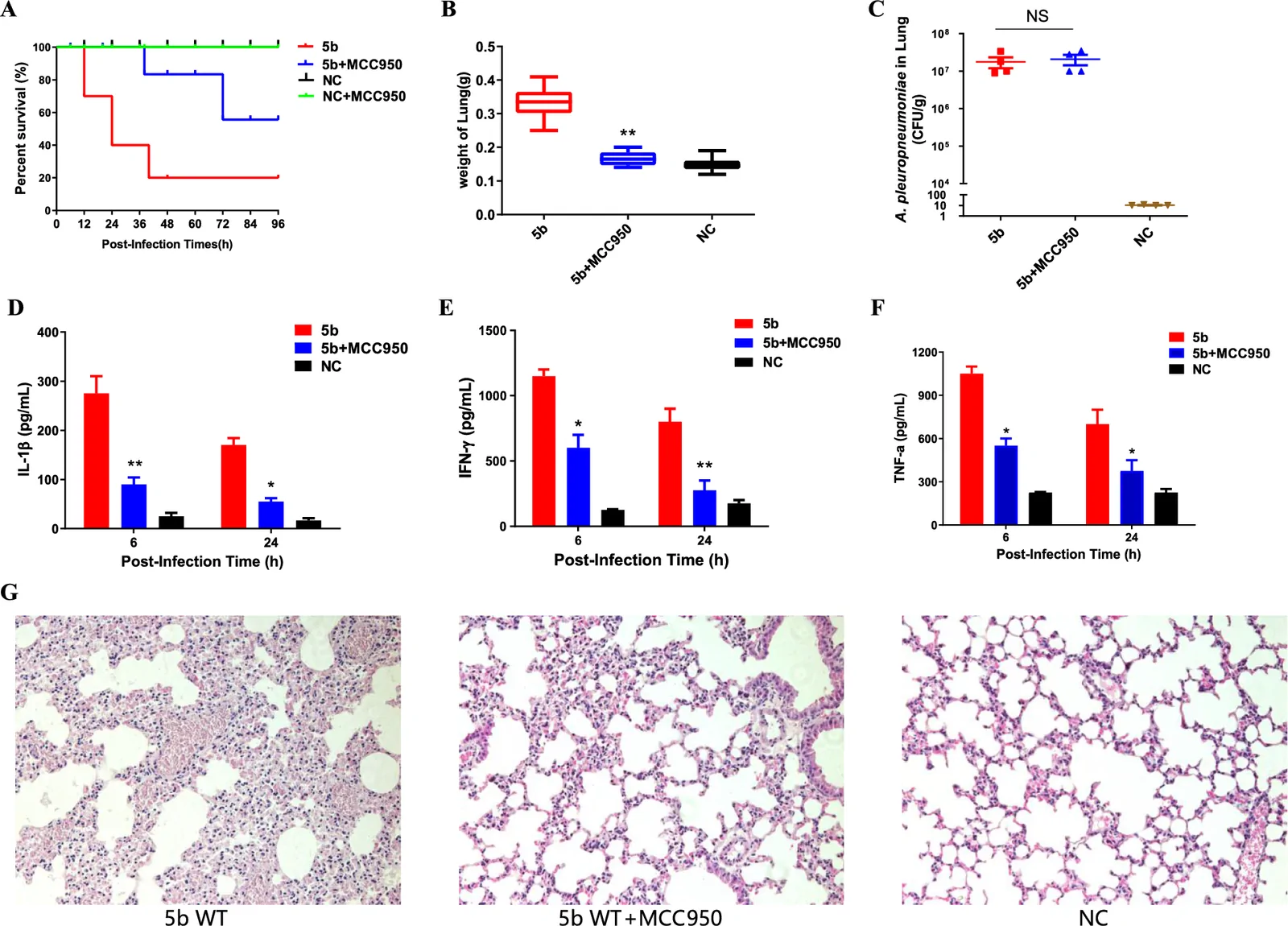
***Actinobacillus pleuropneumoniae*** **results in lung injury via NLRP3-mediated inflammatory response**. BALB/c mice were infected with 5b WT at an infectious dose of 1 × 10^8^ CFU after the mice were given MCC950 (25 mg/kg, intraperitoneal, 5b+MCC950 group) or 0.9% saline (5b group) for 3 h. NC: negative control to 5b group. **A** Survival rate, **B** lung weight, **C** bacterial lung burden and **D** IL-1β, **E** IFN-γ and **F** TNF-α level in the sera of mice in different groups. **G** Histopathology analysis (H&E staining) of mice from the lungs was shown. Error bars indicate the mean (± SD) of three repeats. The data are representative of results from three independent experiments with similar results. *P < 0.05; **P < 0.05. NS, not significant.

The demonstrated role of the NLRP3 inflammasome in 5b WT-induced inflammatory responses prompted us to investigate whether the NLRP3-mediated inflammatory response contributes to 5b WT-induced lung injury. To this end, we employed MCC950, a specific inhibitor of the NLRP3 inflammasome, in a mouse model of infection (1 × 10^8^ CFU per mouse). MCC950 treatment significantly improved survival rates compared with the vehicle-treated group (80% versus 20%) (Figure 3A). Furthermore, both lung weight (Figure 3B) and bacterial burden in the lungs (Figure 3C) were reduced in MCC950-treated mice. ELISA analysis also demonstrated that serum levels of pro-inflammatory cytokines (IL-1β, IFN-γ, and TNF-α) (Figure 3D–F) were significantly lower in the MCC950-treated group at both 6 and 24 hpi.

Histological analysis revealed that 5b WT-infected mice exhibited severe lung lesions characterized by alveolar rupture, pulmonary congestion, and extensive infiltration of inflammatory cells into the pulmonary interstitium, alveoli, and peribronchial regions—pathological features consistent with bronchopneumonia (Figure 3G). In contrast, MCC950-treated mice displayed relatively milder lung pathology with reduced interstitial thickening; inflammatory cells were primarily localized around the bronchi, with minimal alveolar infiltration (Figure 3G).

Collectively, these findings demonstrate that 5b WT induces lung injury through activation of the NLRP3-mediated inflammatory response.

### The Adh domain is the key region for the activation of NLRP3-mediated inflammatory responses during *A. pleuropneumoniae* infection

**Figure 4:**
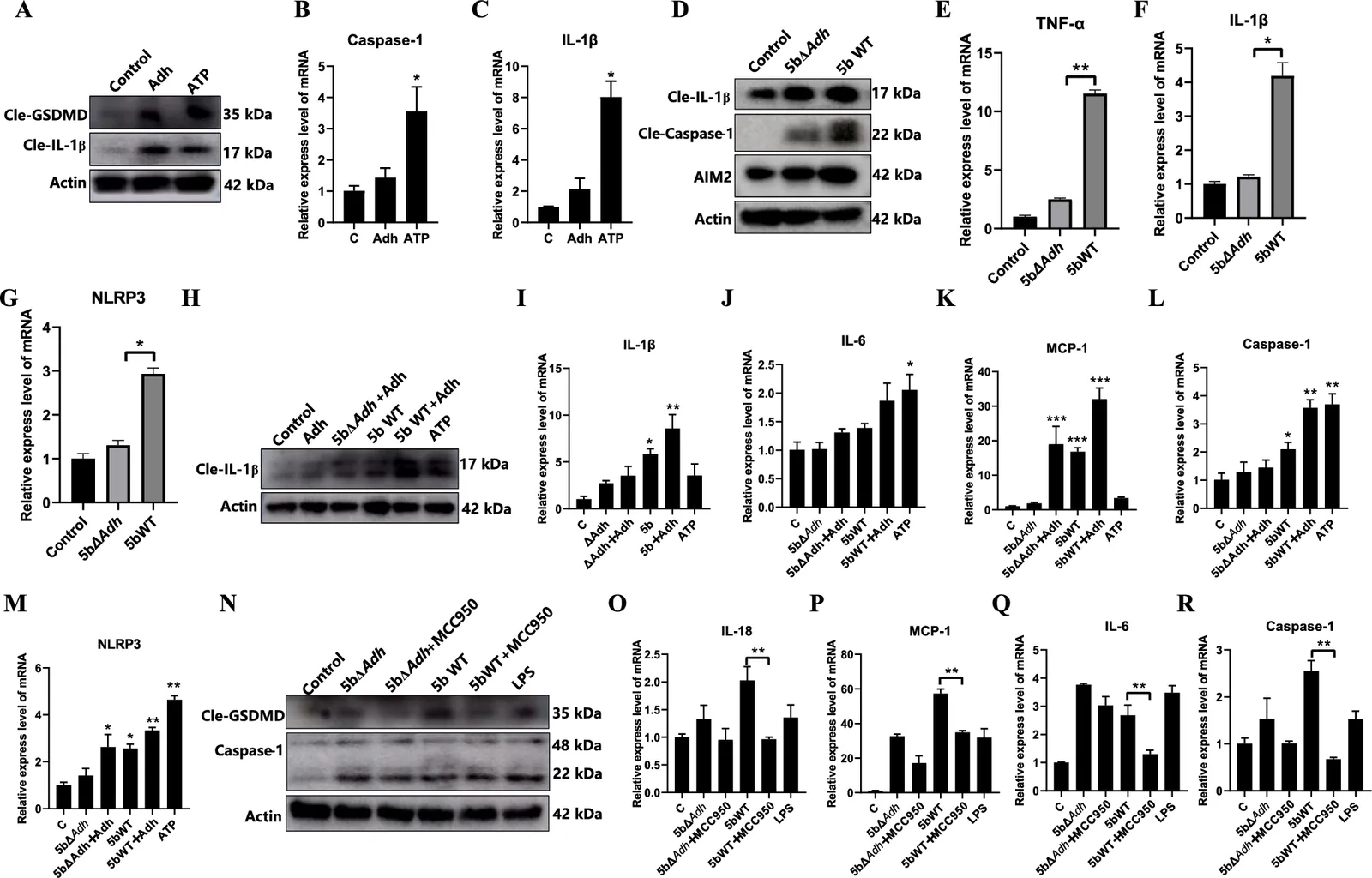
**The Adh adhesin domain is the key region for the activation of NLRP3-mediated inflammatory responses during** ***A. pleuropneumoniae infection***. **A** PAMs were treated with ATP or Adh for 12 h. Untreated group of cells were used as control. Then, the expression of cle-GSDMD and cle-IL-1β in the cells were analyzed by western blot (**A**), and caspase−1 and IL-1β mRNA were further detected by qRT-PCR (**B** and **C**). PAMs were infected with 5b WT or 5b Δ*Adh* and then cultured in contained or not contained Adh protein culture system for 12 h. Untreated group of cells were used as control. Then, the expression of cle-IL-1β, cle-caspase−1 and AIM2 in the cells were analyzed by western blot (**D**), and IL-1β and NLRP3 mRNA were further detected by qRT-PCR (**E**–**G**). PAMs were co-treated with Adh protein and ATP; or infected with 5b WT or 5b ΔAdh with or without Adh protein complementation.12 h later, the expression of cle-IL-1β in the cells were analyzed by western blot (**H**), and IL-1β, IL-6, MCP-1, Caspase−1 and NLRP3 mRNA were further detected by qRT-PCR (**I**–**M**). 5b WT or 5b Δ*Adh* were used to infect PAMs that were pretreated with MCC950. Untreated group of cells were used as control. Then, the expression of cle-GSDMD, caspase−1 and cle-caspase−1 in the cells were analyzed by western blot (**N**), and IL-18, MCP-1, IL-6, and caspase−1 mRNA were further detected by qRT-PCR (**O**–**R**). Error bars indicate the mean (± SD) of three repeats. The data are representative of results from three independent experiments with similar results. *P < 0.05; **P < 0.05. ***P < 0.01.NS, not significant.

We previously identified Adh, located in the head region of the trimeric autotransporter adhesin Apa1 in *A. pleuropneumoniae*, as a critical functional domain that enhances pathogenicity and activates inflammatory responses [20, 21, 33]. To determine whether Adh is responsible for NLRP3-mediated inflammatory responses, we assessed NLRP3 inflammasome-related markers in PAMs pre-treated with purified Adh protein using qRT-PCR and western blotting. Pre-incubation with Adh protein or ATP (positive control) significantly increased GSDMD and IL-1β cleavage (Figure 4A and Additional file 2) and elevated levels of caspase−1 (Figure 4B) and IL-1β (Figure 4C) compared with the control.

To further define the role of Adh in *A. pleuropneumoniae*-induced NLRP3 activation, we infected PAMs with an Adh deletion mutant (5bΔ*Adh*) and compared outcomes with the wild-type (5b WT) strain. 5bΔ*Adh* infection significantly reduced levels of mature caspase−1 and IL-1β (Figure 4D and Additional file 2) and decreased expression of TNF-α, NLRP3 and IL-1β (Figure 4E–G) compared with the 5b WT strain. Subsequently, we performed a complementation assay by treating 5b Δ*Adh*-infected PAMs with purified Adh protein to further assess its role in NLRP3-mediated inflammatory responses. The reduced levels of mature IL-1β (Figure 4H and Additional file 2) and mRNA levels of IL-1β, IL-6, MCP-1, caspase−1 and NLRP (Figure 4I–M) observed in the 5b Δ*Adh*-infected group were restored upon Adh complementation. Additionally, MCC950 further decreased levels of mature caspase−1 and GSDMD (Figure 4N and Additional file 2) and expression of IL-18, MCP-1, IL-6 and caspase−1 (Figure 4O–R) in 5b Δ*Adh*-infected PAMs, implying that other virulence factors in *A. pleuropneumoniae* also mediate NLRP3-mediated inflammatory responses.

Taken together, these data demonstrate that the Adh domain is a key, but not exclusive, region for NLRP3 inflammasome activation during *A. pleuropneumoniae* infection.

### Adh promotes the NLRP3-mediated inflammatory response via OR5M11

**Figure 5:**
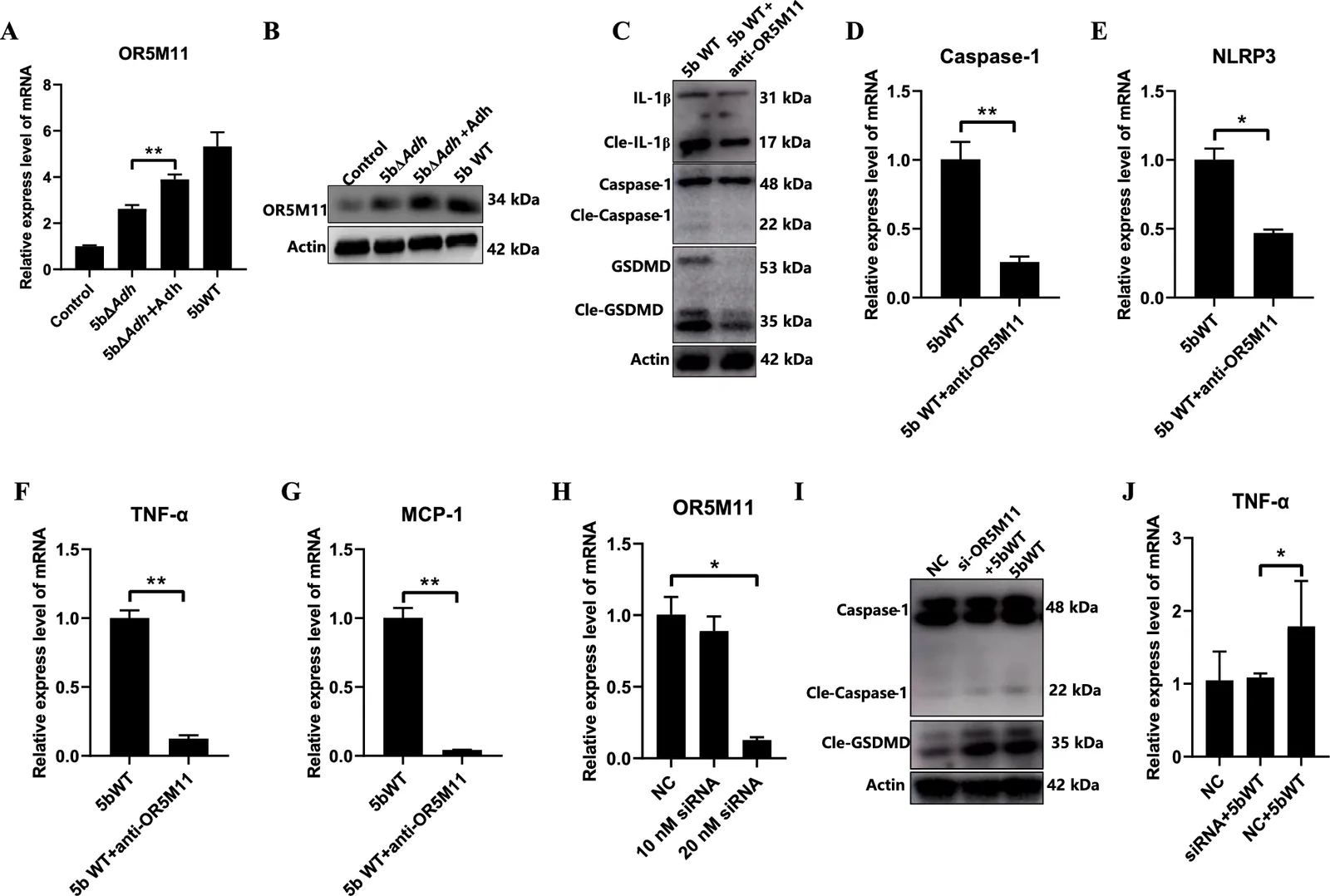
**Specific antibody blockade, or knockdown, of OR5M11 can both inhibit NLRP3-mediated inflammatory responses**. **A** and **B** PAMs were infected with 5b Δ*Adh* and then cultured in contained or not contained Adh protein culture system for 12 h. 5b WT infection PAMs as positive group. The expression of OR5M11 mRNA (**A**) in the cells were analyzed by RT-qPCR, and protein expression was further detected by western blot (**B**). 5b WT were used to infect PAMS that were pretreated with specific primary antibody against OR5M11. Only 5b WT infected cells without any other treatment were used as control. The expression of IL-1β, cle-IL-1β, caspase−1, cle-caspase−1, GSDMD and cle-GSDMD in the cells were analyzed by western blot (**C**), and caspase−1, NLRP3, TNF-α and MCP-1 mRNA were further detected by qRT-PCR (**D**–**H**). The determination of OR5M11 knockdown efficiency. **P**–**T** PAMs were transfected with nonspecific control siRNA (NC) or siRNA against OR5M11 (si-OR5M11) for 12 h and then infected with 5b WT at an MOI of 10 for 12 h. Then, the expression of caspase−1, cle-caspase−1 and cle-GSDMD in the cells were analyzed by western blot (**I**), and TNF-α mRNA (**J**) were further detected by qRT-PCR. Error bars indicate the mean (± SD) of three repeats. The data are representative of results from three independent experiments with similar results. *P < 0.05; **P < 0.05. NS, not significant.

Our previous studies demonstrated that Adh enhances *A. pleuropneumoniae* pathogenicity and regulates inflammatory responses by binding to OR5M11 [20]. To investigate the role of OR5M11 in Adh-induced NLRP3 activation during *A. pleuropneumoniae* infection, we first examined OR5M11 expression in PAMs infected with different bacterial strains using qRT-PCR and western blotting. qRT-PCR analysis revealed that the 5b WT strain significantly increased OR5M11 mRNA levels compared with the control group (Figure 5A). Notably, although the 5b Δ*Adh*-infected group showed reduced expression, this reduction was partially restored upon Adh complementation (Figure 5A). Consistent with these findings, western blot analysis showed similar trends (Figure 5B and Additional file 2).

Next, to determine whether OR5M11 contributes to NLRP3-mediated inflammatory responses, we pre-treated PAMs with an OR5M11-specific antibody prior to 5b WT infection. Our data showed that antibody treatment inhibited not only the maturation of NLRP3 inflammasome–related proteins, including cleaved IL-1β (Figure 5C and Additional file 2), but also the upregulation of inflammatory cytokines such as caspase−1, NLRP3, TNF-α and MCP-1 (Figure 5D–H) in 5b WT-infected PAMs.

Additionally, we knocked down OR5M11 in PAMs via RNA interference to further evaluate its role in NLRP3 inflammasome activation. qRT-PCR was performed to verify knockdown efficiency. Treatment with OR5M11-specific siRNA (si-OR5M11) at various concentrations reduced OR5M11 levels in a dose-dependent manner, with 10 μM producing the most pronounced effect (Figure 5I and Additional file 2). Moreover, si-OR5M11 at 10 μM significantly suppressed the maturation of caspase−1 and GSDMD (Figure 5I and Additional file 2) and inhibited TNF-α expression (Figure 5J).

**Figure 6:**
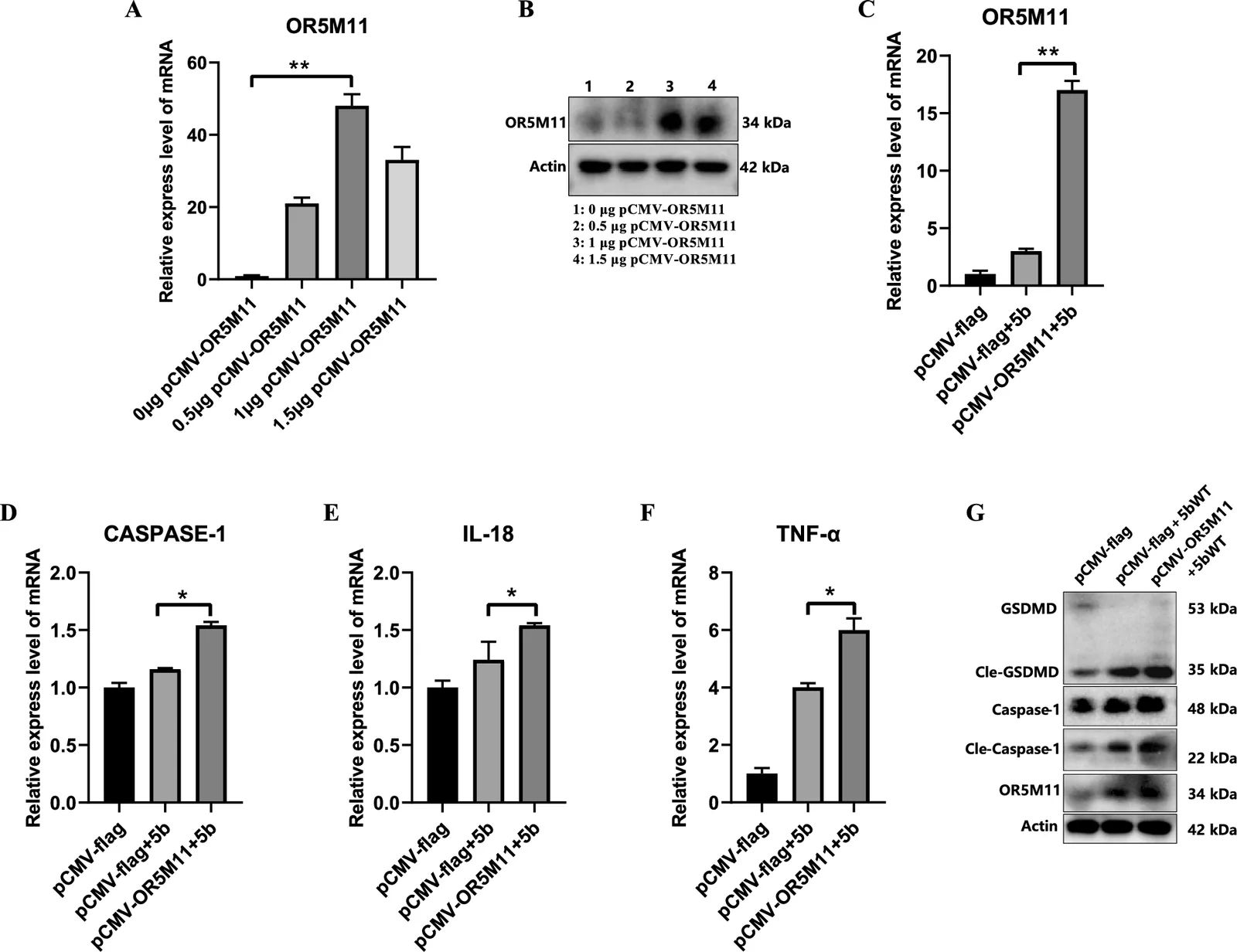
**Overexpression of OR5M11 enhances NLRP3-mediated inflammatory responses**. **A** and **B** The determination of OR5M11 overexpression efficiency. PAMs were transfected with 0, 0.5, 1.0, and 2.0 μg of pCMV-OR5M11 respectively. 24 h later, the expression of OR5M11 mRNA (**A**) in the cells were analyzed by RT-qPCR, and protein expression was further detected by western blot (**B**). PAMs were transfected with pCMV-OR5M11, and 24 h later, the cells were infected with 5b WT. 12 h later, the mRNA level of OR5M11 (**C**), caspase−1 (**D**), IL-18 (**E**), TNF-α (**G**) were detected by qRT-PCR, and the protein expression of IL-1β, cle-IL-1β, caspase−1, cle-caspase−1, GSDMD, cle-GSDMD, and OR5M11 in the cells were analyzed by western blot (**G**). Error bars indicate the mean (± SD) of three repeats. The data are representative of results from three independent experiments with similar results. *P < 0.05; **P < 0.05. NS, not significant.

In parallel, we transfected PAMs with a pCMV plasmid expressing OR5M11 (pCMV-OR5M11) or an empty vector (pCMV-N1). After 24 h, qRT-PCR and western blotting were performed to confirm overexpression efficiency. Both mRNA (Figure 6A) and protein (Figure 6B and Additional file 2) levels of OR5M11 were significantly elevated in PAMs transfected with the overexpression plasmid. As expected, OR5M11 overexpression further enhanced the mRNA levels of OR5M11, caspase−1, IL-18 and TNF-α, (Figure 6C–F) and promoted the maturation of GSDMD, caspase−1, and IL-1β proteins (Figure 6G and Additional file 2) during 5b WT infection.

Therefore, we conclude that Adh promotes NLRP3-mediated inflammatory responses by engaging OR5M11.

### OR5M11 participates in the NLRP3-mediated inflammatory response by promoting mitochondrial DNA release

**Figure 7:**
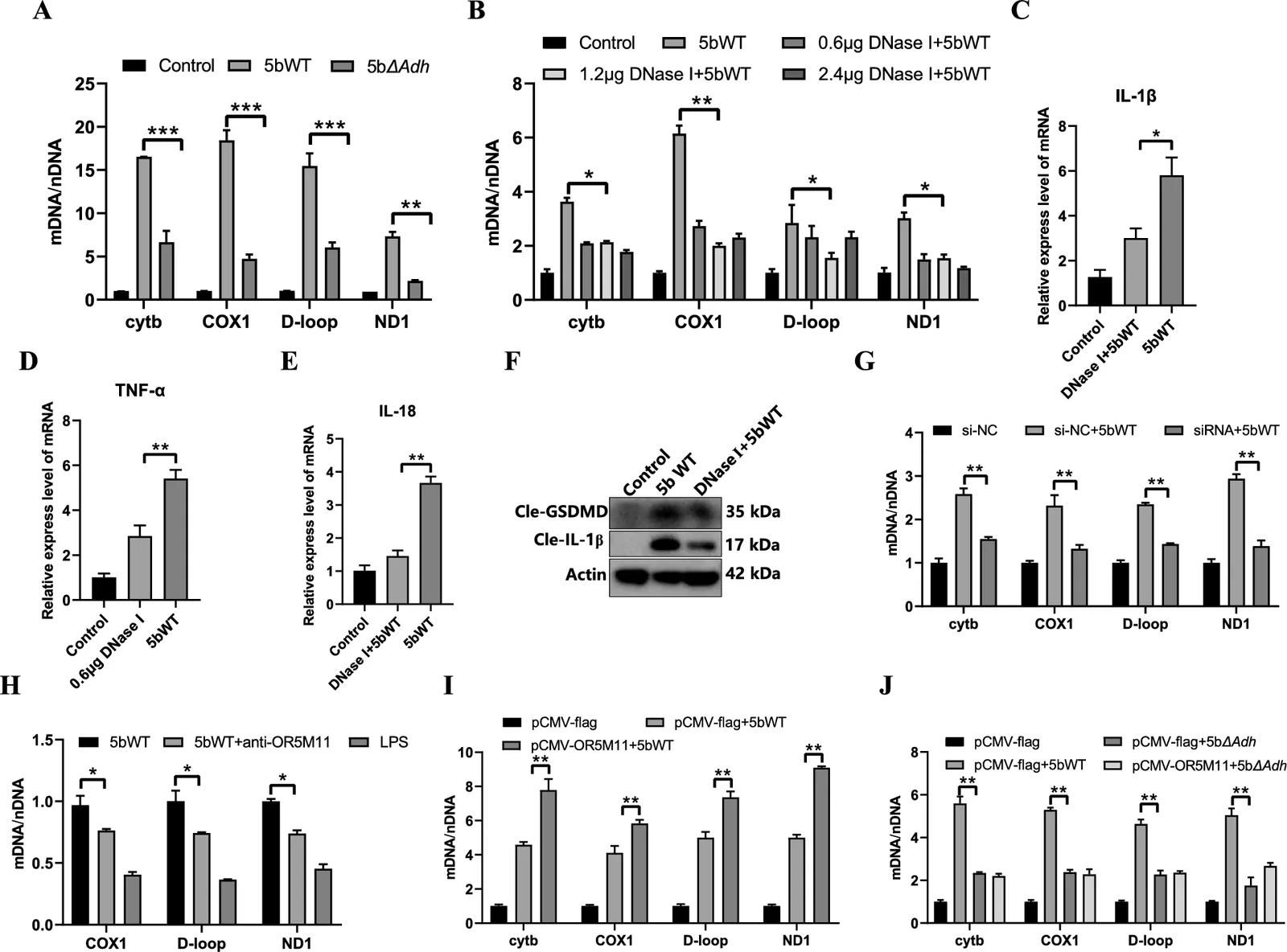
**Mitochondrial DNA release participates in OR5M11 facilitated NLRP3-mediated inflammatory responses**. **A** PAMs were infected with 5b WT or 5b Δ*Adh.*12 h later, the mtDNA released in cytoplasm were extracted and the quantified by qRT-PCR. **B** PAMs were first transfected respectively with 0.6, 1.2 and 2.4 μg of DNase I for 24 h, then infected with 5b-WT for 12 h. Then, the mtDNA released in cytoplasm were extracted and the quantified by qRT-PCR. **C**–**H** PAMs were transfected with 1.2 μg of DNase I for 24 h, then infected with 5b-WT for 12 h. Then, IL-18, TNF-α and IL-1β mRNA expression in the cells were analyzed by qRT-PCR (**C**–**E**), and the protein expression of cle-IL-1β and cle-GSDMD were further detected by western blot (**F**). **G** PAMs were transfected with nonspecific control siRNA (NC) or siRNA against OR5M11 (si-OR5M11) for 12 h and then infected with 5b WT at an MOI of 10 for 12 h. Then, the mtDNA released in cytoplasm were extracted and the quantified by qRT-PCR. **H** PAMs were pretreated with or not with specific primary antibody against OR5M11 post-infection with 5b WT for 12 h. Then, the mtDNA released in cytoplasm were extracted and the quantified by qRT-PCR. **I** PAMs were transfected with pCMV-OR5M11, and 24 h later, the cells were infected with 5b WT. 12 h later, the mtDNA released in cytoplasm were extracted and the quantified by qRT-PCR. **J** PAMs were transfected with empty vector or pCMV-OR5M11, 24 h later, infected with 5b WT or 5b Δ*Adh* for 12 h. Then, the mtDNA released in cytoplasm were extracted and the quantified by qRT-PCR. Error bars indicate the mean (± SD) of three repeats. The data are representative of results from three independent experiments with similar results. *P < 0.05; **P < 0.05. NS, not significant.

It has previously been shown that Adh affects mitochondrial homeostasis, as evidenced by reduced mitochondrial membrane potential (MMP) in PAMs [27], indicating mitochondrial dysfunction and excessive opening of the mitochondrial permeability transition pore (mPTP). As a major mitochondrial damage-associated molecular pattern (DAMP), mtDNA leaking into the cytoplasm during mitochondrial dysfunction can directly engage pattern recognition receptors (PRRs) to enhance pro-inflammatory responses [34]. Given the interaction between Adh and OR5M11, we hypothesized that Adh triggers NLRP3-mediated inflammatory responses through OR5M11-mediated mtDNA release. To test this, we first investigated whether *A. pleuropneumoniae* infection induces mtDNA release into the cytosol by quantifying cytosolic mtDNA using qRT-PCR. The mtDNA content (including cyt b, COX I, D-loop, and ND1) in the cytoplasm of 5b WT-infected PAMs was significantly increased compared with that in control cells (Figure 7A). Notably, although infection with the 5b Δ*Adh* strain also promoted mtDNA release, this promotion was less pronounced than that observed in the 5b WT-infected group, indicating that Adh contributes to mtDNA release (Figure 7A).

Subsequently, we explored the role of mtDNA released from mitochondria in NLRP3-mediated inflammatory responses by using DNase I to degrade cytosolic mtDNA in *A. pleuropneumoniae*-infected cells and then assessed the effects on NLRP3 inflammasome activation and inflammatory cytokine expression. qRT-PCR results revealed that DNase I decreased not only cytosolic mtDNA levels (Figure 7B) but also the excessive expression of inflammasome cytokines, such as IL-1β, TNF-α and IL-18 (Figure 7C–E), induced by 5b WT infection. Additionally, NLRP3 inflammasome–related proteins, including mature GSDMD, IL-1β, and AIM2, were downregulated in DNase I-treated PAMs during 5b WT infection (Figure 7F and Additional file 2).

To investigate the involvement of OR5M11 in mediating mtDNA release, OR5M11 was knocked down using siRNA, and qRT-PCR was performed to measure mtDNA levels. The data indicated that the release of mtDNA (cyt b, COX I, D-loop, and ND1) into the cytosol was significantly inhibited by knocking down OR5M11 during 5b WT infection in PAMs (Figure 7G). Similar results were obtained in PAMs pretreated with an OR5M11-specific antibody (Figure 7H). Conversely, overexpression of OR5M11 by transfection with a pCMV-OR5M11 plasmid further increased the level of mtDNA (cyt b, COX I, D-loop, and ND1) released into the cytosol during 5b WT infection (Figure 7I). Importantly, overexpression of OR5M11 in 5b Δ*Adh*-infected PAMs failed to further promote mtDNA release (Figure 7J), indicating that Adh is essential for OR5M11-mediated mtDNA release; in other words, cooperation between Adh and OR5M11 promotes mtDNA release.

Collectively, our results demonstrate that OR5M11 cooperates with Adh to facilitate *A. pleuropneumoniae*-triggered mtDNA release, which triggers NLRP3-mediated inflammatory responses.

### Adh is a critical virulence factor that contributes to the lung pathogenicity of *A. pleuropneumoniae*

Given that Adh and OR5M11 participate in *A. pleuropneumoniae*-induced, NLRP3-mediated inflammatory responses in vitro, we sought to explore the contributions of these bacterial and host factors to *A. pleuropneumoniae* pathogenicity, particularly pulmonary inflammatory injury. Therefore, we established four experimental groups in a mouse model: a negative control group (NC), a 5b WT-infected group (5b), a 5b Δ*Adh*-infected group (5b Δ*Adh*), and a complementation group in which mice received intraperitoneal Adh injection prior to infection with the 5b Δ*Adh* strain (5b cΔAdh), to verify the role of Adh in *A. pleuropneumoniae* pathogenicity.

**Figure 8:**
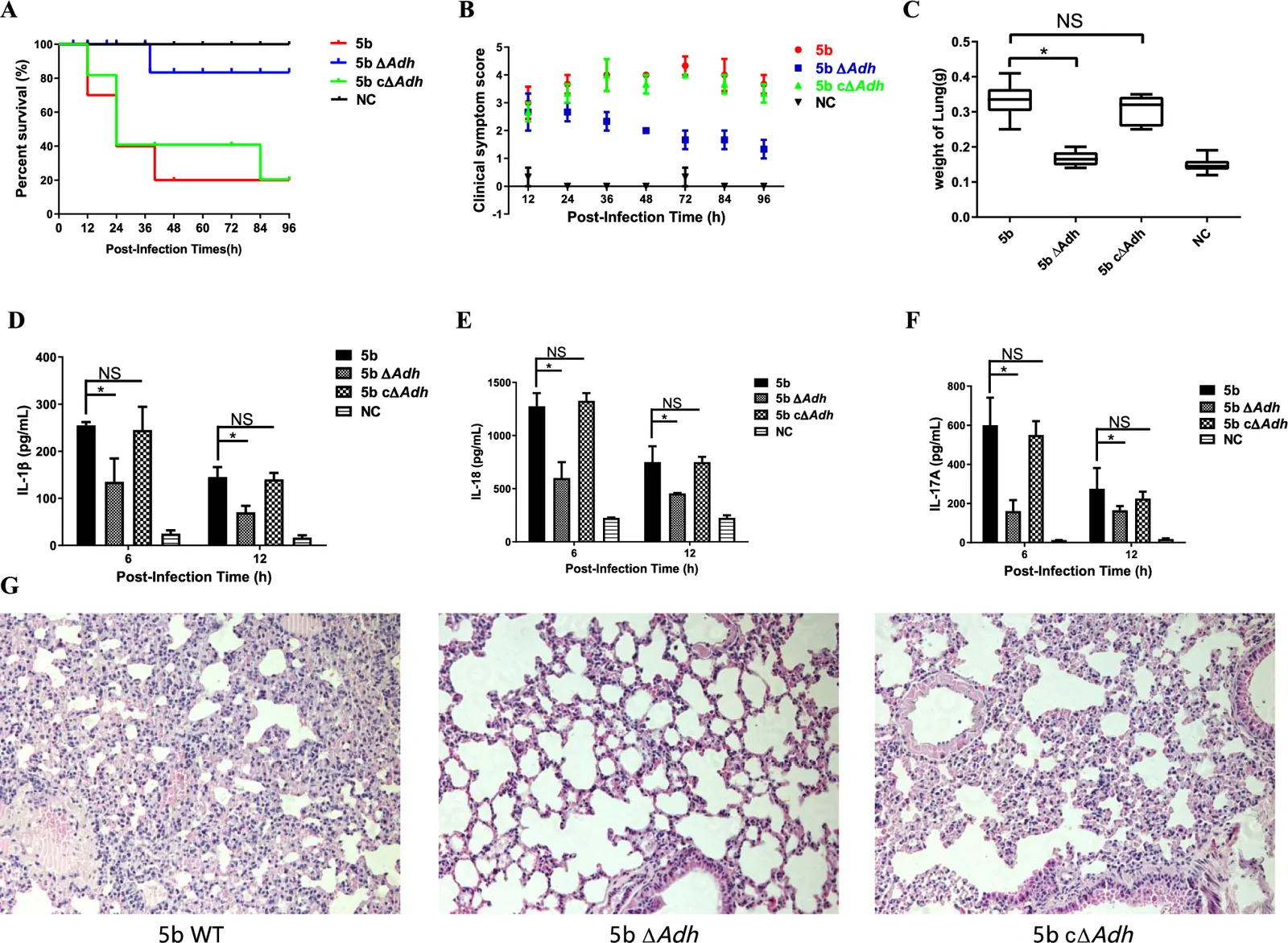
**Verification the role of Adh in the lung pathogenicity of** ***A. pleuropneumoniae*** **in vivo**. Per BALB/c mice was infected with 5b WT (5b group) or 5b Δ*Adh* (5b Δ*Adh* 5b group) at an infectious dose of 1 × 10^8^ CFU, or infected with 5b Δ*Adh* after injected intraperitoneally with Adh (5b *c*Δ*Adh*5b group). NC: negative control group. **A** Survival rate, **B** clinical symptom score, **C** lung weight and **D** IL-1β, **E** IL-18 and **F** IL-17A level in the sera of mice in different groups. **G** Histopathology analysis (H&E staining) of mice from the lungs was shown. Error bars indicate the mean (± SD) of three repeats. The data are representative of results from three independent experiments with similar results. *P < 0.05; NS, not significant.

As shown in Figure 8A, the survival rate of the 5b group was significantly lower than that of the 5b Δ*Adh* group (20% versus 80%, respectively). Notably, the 5b cΔAdh group exhibited reduced survival between 42 and 84 hpi compared with the 5b Δ*Adh* group; however, survival ultimately equaled that of the 5b group. Clinical scores showed a similar trend to the survival data (Figure 8B). Lungs from the 5b group were the heaviest, whereas those from the 5b Δ*Adh* group were significantly lighter (Figure 8C). Consistently, serum levels of IL-1β (Figure 8D), IL-18 (Figure 8E), and IL-6 (Figure 8F) in the 5b Δ*Adh* group were significantly lower than those in the 5b group but higher than those in the NC group at 12 and 24 h post-infection. Additionally, histopathological analysis revealed marked inflammatory exudates and widened interstitial spaces in the 5b group compared with the 5b Δ*Adh* group (Figure 8G). Furthermore, extensive red blood cell infiltration in the alveoli of the 5b group indicated severe pulmonary hemorrhage (Figure 8G). The 5b cΔ*Adh* group exhibited intermediate pathological severity between the 5b and 5b Δ*Adh* groups (Figure 8G).

### Targeting OR5M11 protects against lung injury induced by *A. pleuropneumoniae*

**Figure 9:**
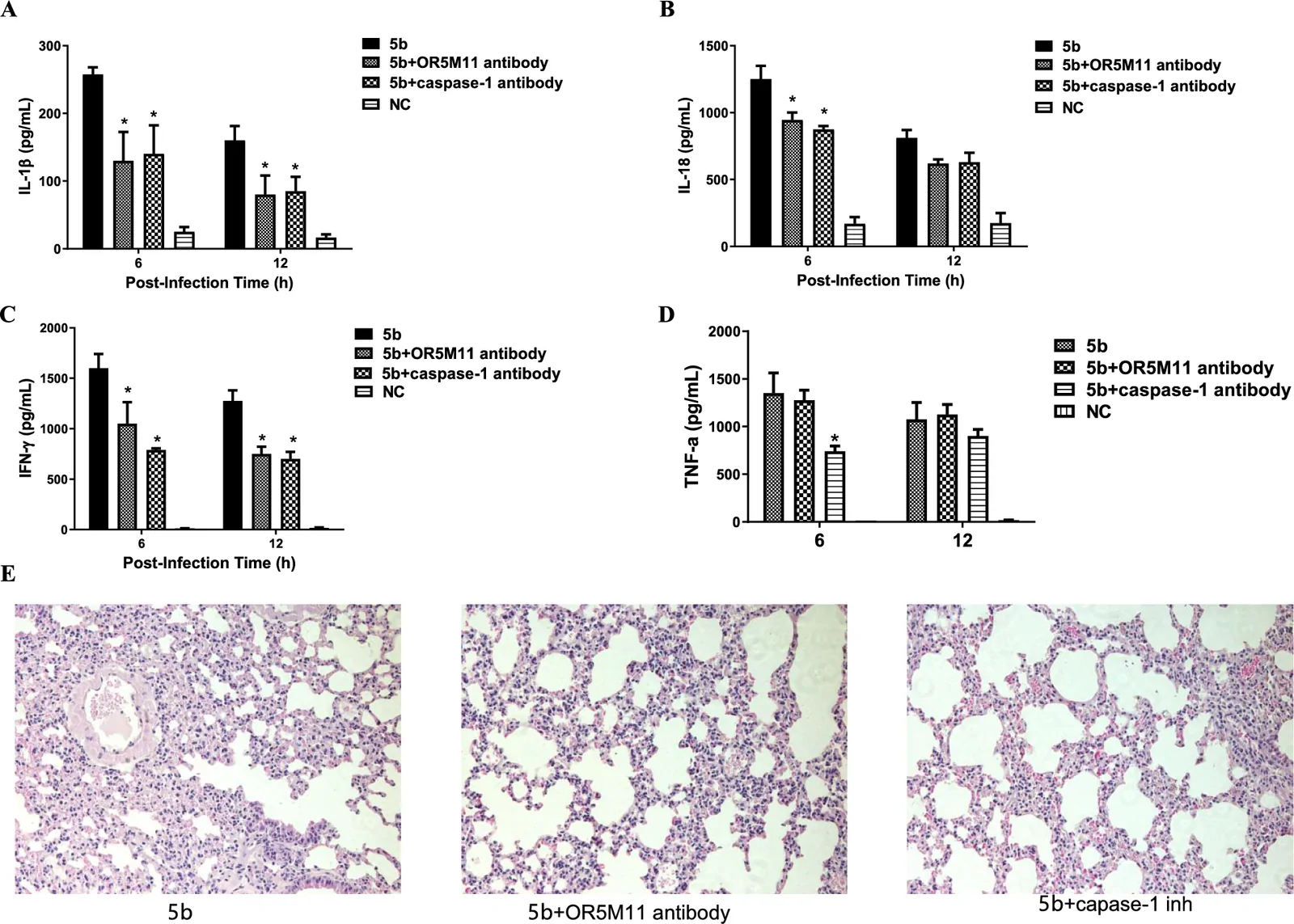
**Determination the role of OR5M11 in the lung pathogenicity of** ***A. pleuropneumoniae*** **in vivo.** Mice were pretreated with specific primary antibody against OR5M11 or with Z-YVAD-FMK (caspase−1 inhibitor) post-infection with 5b WT. NC: negative control group. **A** IL-1β, **B** IL-18, **C** IFN-γ and **D** TNF-α level in the sera of mice in different groups. **E** Histopathology analysis (H&E staining) of mice from the lungs was shown. Error bars indicate the mean (± SD) of three repeats. The data are representative of results from three independent experiments with similar results. *P < 0.05; NS, not significant.

The role of OR5M11 in the pathogenicity of *A. pleuropneumoniae* was also investigated by intraperitoneal administration of an OR5M11-specific antibody to mice. ELISA results showed that the OR5M11-specific antibody significantly decreased serum levels of IL-1β (Figure 9A), IL-18 (Figure 9B), IFN-γ (Figure 9C), and TNF-α (Figure 9D) and alleviated lung injury (Figure 9L, middle panel). Similar results were obtained with a caspase−1-inhibitor, further suggesting that NLRP3-mediated inflammatory responses, particularly activation of the NLRP3 inflammasome, contribute to lung injury during *A. pleuropneumoniae* infection (Figure 9E, right panel). Collectively, these results demonstrate that Adh and OR5M11 are critical bacterial and host factors, respectively, that contribute to the pulmonary pathogenicity of *A. pleuropneumoniae*.

## Discussion

*Actinobacillus pleuropneumoniae*-induced porcine contagious pleuropneumonia is characterized by severe lung damage, including marked congestion and fibrosis. Furthermore, high levels of inflammatory cytokines are present in the lungs of *A. pleuropneumoniae*-infected pigs, accompanied by considerable inflammatory cell infiltration and severe alveolar structural damage, indicative of typical inflammatory lung injury [4, 5, 8]. This is a novel study to elucidate the underlying mechanism by which *A. pleuropneumoniae* infection induces pathological inflammation in the lung; we demonstrate that the NLRP3-mediated inflammatory response contributes to the pulmonary pathogenicity of *A. pleuropneumoniae*. However, as a major pathogen of the Porcine Respiratory Disease Complex (PRDC), *A. pleuropneumoniae* frequently co-occurs with other pathogens, particularly viruses, thereby exacerbating lung lesions [35, 36]. Therefore, investigating the role of the NLRP3-mediated inflammatory response during co-infection with *A. pleuropneumoniae* and other pathogens represents an important direction for future research.

Two trimeric autotransporter adhesins (TAAs), designated Apa1 and Apa2, were identified in *A. pleuropneumoniae* in 2011 and 2012, respectively, and mediate autoagglutination and host cell adherence [18, 19]. Subsequently, amino acids 68–612 in the head region of Apa1, designated Adh, were demonstrated to constitute the primary functional domain mediating *A. pleuropneumoniae* adhesion [21]. In addition to mediating adherence, Adh binds to OR5M11 to regulate proinflammatory cytokine expression, particularly IL-8, which plays a critical role in neutrophil recruitment and contributes to lung inflammation and fibrosis when highly expressed [20]. The present study elucidates that the interaction between Adh and OR5M11 drives excessive activation of the NLRP3-mediated inflammatory response, ultimately resulting in lung damage. Notably, the Adh–OR5M11 interaction represents a critical process that enhances pulmonary pathogenicity. Therefore, determining the structure of the Adh–OR5M11 complex and designing small-molecule inhibitors targeting this interaction will be crucial for preventing and treating lung inflammatory damage caused by *A. pleuropneumoniae* infection.

Olfactory receptors (ORs), which belong to class A G protein-coupled receptors (GPCRs), constitute the most important chemosensory receptor family responsible for olfaction in the nasal olfactory epithelium [37]. Interestingly, ORs are also expressed in macrophages, including alveolar macrophages, where they can modulate inflammatory responses and have been implicated in various lung diseases [38, 39]. In our previous study, we demonstrated that OR5M11, an OR family member, is expressed in porcine alveolar macrophages and mediates the release of inflammatory cytokines, contributing to *A. pleuropneumoniae* pathogenicity through its interaction with Adh [20]. However, the mechanism by which OR5M11 regulates *A. pleuropneumoniae*-induced inflammatory responses remains unknown. Recently, an important study revealed that olfactory receptor 2 in vascular macrophages drives atherosclerosis through NLRP3-dependent IL-1 production [40]. Here, we demonstrate that OR5M11 also participates in NLRP3-mediated inflammatory responses, ultimately contributing to lung damage during *A. pleuropneumoniae* infection through the aberrant release of mitochondrial DNA. Whereas, how does OR5M11 function in causing abnormal release of mitochondrial DNA? As known, in the process of G protein-coupled receptors related signal transduction, an influx of calcium ions (Ca^2+^) into the cells, which is able to trigger a series of downstream signaling pathways, especially is related to mitochondrial homeostasis [40–42]. Additionally, ORs can also activate the phosphatidylinositol 3-kinase (PI3K)-Akt pathway and the mitogen-activated protein kinase (MAPK) pathway, which regulate some biological process of cells [43, 44]. Therefore, we make a hypothesis that influx of calcium ions (Ca^2+^), and PI3K-Akt and MAPK pathway may be involved in OR5M11-mediated mitochondrial DNA release during *A. pleuropneumoniae* infection. But this need to be further studied.

Mitochondrial DNA (mtDNA) is a major mitochondrial damage-associated molecular pattern (DAMP) that escapes from stressed mitochondria into the cytoplasm and provokes inflammation, particularly NLRP3 inflammasome activation [45–47]. NLRP3 inflammasome activation follows a two-step process: priming and activation [48]. Priming is initiated by pattern recognition receptors (PRRs) that sense pathogen-associated molecular patterns (PAMPs) or danger-associated molecular patterns (DAMPs), leading to NF-κB-mediated transcription of NLRP3 and pro-IL-1β. Activation involves NLRP3 inflammasome assembly, caspase−1 activation, and IL-1β maturation. mtDNA plays a key role in this two-step activation of the NLRP3 inflammasome. On the one hand, cytosolic mtDNA, sensed by cyclic GMP-AMP (cGAMP) synthase (cGAS)—a PRR—activates the cGAS-STING pathway, which subsequently promotes the transcription of genes encoding NLRP3 inflammasome activation–related proteins, such as IL-1β and NLRP3, through NF-κB activation [49]^.^ On the other hand, mtDNA binds to cytosolic NLRP3, thereby triggering inflammasome activation [37]. In the present study, we found that mtDNA released from *A. pleuropneumoniae*-infected PAMs participates in NLRP3-mediated inflammatory responses. However, the roles of the cGAS-STING pathway and the direct binding of mtDNA to NLRP3 in the NLRP3-mediated inflammatory response triggered by OR5M11-promoted mtDNA release during *A. pleuropneumoniae* infection were not investigated in this study and require further verification.

**Figure 10:**
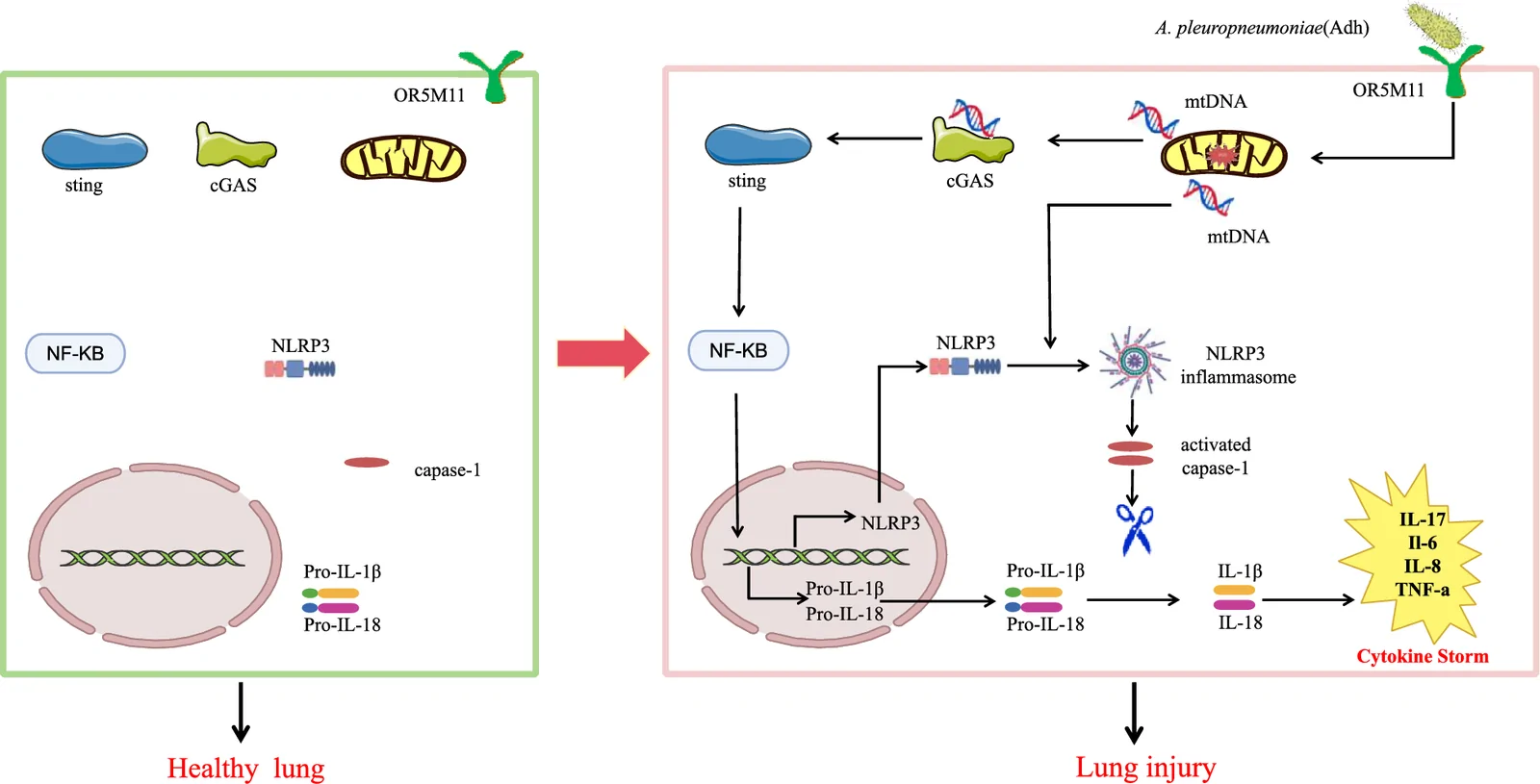
**A schematic model illustrating how the** ***A. pleuropneumoniae*** **Adh protein engages OR5M11 to enhance pulmonary pathogenicity by facilitating the NLRP3-mediated inflammatory response**. During infection, Adh binding to OR5M11 triggers mitochondrial DNA release, leading to dysregulated NLRP3-mediated inflammatory responses that ultimately exacerbate lung injury.

A schematic model illustrating how the NLRP3-mediated inflammatory response, activated through the interaction of Adh with OR5M11, contributes to the pulmonary pathogenicity of *A. pleuropneumoniae* is presented in Figure 10. In this model, Adh binding to OR5M11 triggers mtDNA release, resulting in dysregulated NLRP3-mediated inflammatory responses that ultimately exacerbate lung injury during *A. pleuropneumoniae* infection. The findings presented herein advance our understanding of *A. pleuropneumoniae* pathogenesis and may inform the development of attenuated vaccines against this pathogen. It must, however, be recognized that, although the mechanism by which the A. pleuropneumoniae virulence factor Adh engages OR5M11 to activate the NLRP3 inflammasome and thereby induce lung inflammatory injury has been characterized in this study, the use of a murine model is unlikely to fully reflect the pathophysiological processes that occur in the natural host, the pig, due to interspecies differences in pulmonary architecture, immune cell composition, and cytokine dynamics. An in vivo study in the natural host, the pig, relating clinical lung pathology to activation of the Adh–OR5M11–NLRP3 axis during A. pleuropneumoniae infection is required to convincingly address this question.

## Supplementary Information

**Additional file 1.** **The PCR primer pairs used in this study.**

**Additional file 2.** **The relative quantification of indicated protein levels.**

## Data Availability

The data that supported the findings of this study are available from the authors upon reasonable request.

## References

[1] Li N, Zhu J, Chen P, Bao C, Wang J, Abdelaal T, Chen D, Zhu S, Wang W, Mao J, Scicluna BP, Koning F, Li F, Lei L (2023) High-dimensional analysis reveals an immune atlas and novel neutrophil clusters in the lungs of model animals with *Actinobacillus pleuropneumoniae*-induced pneumonia. Vet Res 54:76 10.1186/s13567-023-01207-4 PMC10500746 37705063

[2] Soto Perezchica MM, Guerrero Barrera AL, Avelar Gonzalez FJ, Quezada Tristan T, Macias Marin O (2023) *Actinobacillus pleuropneumoniae*, surface proteins and virulence: a review. Front Vet Sci 10:1276712 10.3389/fvets.2023.1276712 PMC10720984 38098987

[3] Ma Q, Sun C, Yang F, Wang L, Qin W, Xia X, Feng X, Du C, Gu J, Han W, Lei L (2015) Macrophages largely contribute to heterologous anti-*Propionibacterium acnes* antibody-mediated protection from *Actinobacillus pleuropneumoniae* infection in mice. Microbiol Immunol 59:166–173 10.1111/1348-0421.12240 25644652

[4] Bosse JT, Janson H, Sheehan BJ, Beddek AJ, Rycroft AN, Kroll JS, Langford PR (2002) *Actinobacillus pleuropneumoniae*: pathobiology and pathogenesis of infection. Microbes Infect 4:225–235 10.1016/s1286-4579(01)01534-9 11880056

[5] Sassu EL, Bosse JT, Tobias TJ, Gottschalk M, Langford PR, Hennig-Pauka I (2018) Update on *Actinobacillus pleuropneumoniae*-knowledge, gaps and challenges. Transbound Emerg Dis 65:72–90 10.1111/tbed.12739 29083117

[6] Czyzewska-Dors E, Dors A, Kwit K, Stasiak E, Pomorska-Mol M (2017) Pig lung immune cytokine response to the swine influenza virus and the *Actinobacillus pleuropneumoniae* infection. J Vet Res 61:259–265 10.1515/jvetres-2017-0036 PMC5894434 29978082

[7] Bao C, Liu B, Zhu R, Xiao J, Li Z, Jiang H, Wang B, Langford PR, Fei R, Li N, Lei L (2021) IFN-gamma(-/-) mice resist *Actinobacillus pleuropneumoniae* infection by promoting early lung IL-18 release and PMN-I accumulation. Infect Immun 89:00069–21 10.1128/IAI.00069-21 PMC8316061 33685942

[8] Bao C, Jiang H, Zhu R, Liu B, Xiao J, Li Z, Chen P, Langford PR, Zhang F, Lei L (2020) Differences in pig respiratory tract and peripheral blood immune responses to *Actinobacillus pleuropneumoniae*. Vet Microbiol 247:108755 10.1016/j.vetmic.2020.108755 32686648

[9] Wang L, Qin W, Ruidong Z, Liu S, Zhang H, Sun C, Feng X, Gu J, Du C, Han W, Langford PR, Lei L (2015) Differential gene expression profiling of *Actinobacillus pleuropneumoniae* during induction of primary alveolar macrophage apoptosis in piglets. Microb Pathog 78:74–86 10.1016/j.micpath.2014.11.017 25435362

[10] Mikula KM, Kolodziejczyk R, Goldman A (2012) *Yersinia* infection tools-characterization of structure and function of adhesins. Front Cell Infect Microbiol 2:169 10.3389/fcimb.2012.00169 PMC3539135 23316485

[11] Totsika M, Wells TJ, Beloin C, Valle J, Allsopp LP, King NP, Ghigo JM, Schembri MA (2012) Molecular characterization of the EhaG and UpaG trimeric autotransporter proteins from pathogenic *Escherichia coli*. Appl Environ Microbiol 78:2179–2189 10.1128/AEM.06680-11 PMC3302597 22286983

[12] Casutt-Meyer S, Renzi F, Schmaler M, Jann NJ, Amstutz M, Cornelis GR (2010) Oligomeric coiled-coil adhesin YadA is a double-edged sword. PLoS One 5:e15159 10.1371/journal.pone.0015159 PMC2999546 21170337

[13] Scarselli M, Serruto D, Montanari P, Capecchi B, Adu-Bobie J, Veggi D, Rappuoli R, Pizza M, Arico B (2006) *Neisseria meningitidis* NhhA is a multifunctional trimeric autotransporter adhesin. Mol Microbiol 61:631–644 10.1111/j.1365-2958.2006.05261.x 16803596

[14] Valle J, Mabbett AN, Ulett GC, Toledo-Arana A, Wecker K, Totsika M, Schembri MA, Ghigo JM, Beloin C (2008) UpaG, a new member of the trimeric autotransporter family of adhesins in uropathogenic *Escherichia coli*. J Bacteriol 190:4147–4161 10.1128/JB.00122-08 PMC2446758 18424525

[15] Szczesny P, Linke D, Ursinus A, Bar K, Schwarz H, Riess TM, Kempf VA, Lupas AN, Martin J, Zeth K (2008) Structure of the head of the *Bartonella* adhesin BadA. PLoS Pathog 4:e1000119 10.1371/journal.ppat.1000119 PMC2483945 18688279

[16] Ruiz-Ranwez V, Posadas DM, Van der Henst C, Estein SM, Arocena GM, Abdian PL, Martin FA, Sieira R, De Bolle X, Zorreguieta A (2013) BtaE, an adhesin that belongs to the trimeric autotransporter family, is required for full virulence and defines a specific adhesive pole of *Brucella suis*. Infect Immun 81:996–1007 10.1128/IAI.01241-12 PMC3584859 23319562

[17] Serruto D, Spadafina T, Scarselli M, Bambini S, Comanducci M, Hohle S, Kilian M, Veiga E, Cossart P, Oggioni MR, Savino S, Ferlenghi I, Taddei AR, Rappuoli R, Pizza M, Masignani V, Arico B (2009) HadA is an atypical new multifunctional trimeric coiled-coil adhesin of *Haemophilus influenzae biogroup aegyptius*, which promotes entry into host cells. Cell Microbiol 11:1044–1063 10.1111/j.1462-5822.2009.01306.x 19290916

[18] Qin W, Wang L, Zhai R, Ma Q, Liu J, Bao C, Zhang H, Sun C, Feng X, Gu J, Du C, Han W, Langford PR, Lei L (2016) Trimeric autotransporter adhesins contribute to *Actinobacillus pleuropneumoniae* pathogenicity in mice and regulate bacterial gene expression during interactions between bacteria and porcine primary alveolar macrophages. Antonie Van Leeuwenhoek 109:51–70 10.1007/s10482-015-0609-x 26494209

[19] Xiao L, Zhou L, Sun C, Feng X, Du C, Gao Y, Ji Q, Yang S, Wang Y, Han W, Langford PR, Lei L (2012) Apa is a trimeric autotransporter adhesin of *Actinobacillus pleuropneumoniae* responsible for autoagglutination and host cell adherence. J Basic Microbiol 52:598–607 10.1002/jobm.201100365 22143982

[20] Wang L, Qin W, Zhang J, Bao C, Zhang H, Che Y, Sun C, Gu J, Feng X, Du C, Han W, Richard PL, Lei L (2016) Adh enhances *Actinobacillus pleuropneumoniae* pathogenicity by binding to OR5M11 and activating p38 which induces apoptosis of PAMs and IL-8 release. Sci Rep 6:24058 10.1038/srep24058 PMC4820727 27046446

[21] Wang L, Qin W, Yang S, Zhai R, Zhou L, Sun C, Pan F, Ji Q, Wang Y, Gu J, Feng X, Du C, Han W, Langford PR, Lei L (2015) The Adh adhesin domain is required for trimeric autotransporter Apa1-mediated *Actinobacillus pleuropneumoniae* adhesion, autoaggregation, biofilm formation and pathogenicity. Vet Microbiol 177:175–183 10.1016/j.vetmic.2015.02.026 25818350

[22] Broz P, Dixit VM (2016) Inflammasomes: mechanism of assembly, regulation and signalling. Nat Rev Immunol 16:407–420 10.1038/nri.2016.58 27291964

[23] Martinon F, Mayor A, Tschopp J (2009) The inflammasomes: guardians of the body. Annu Rev Immunol 27:29–265 10.1146/annurev.immunol.021908.132715 19302040

[24] Rathinam VA, Fitzgerald KA (2016) Inflammasome complexes: emerging mechanisms and effector functions. Cell 165:792–800 10.1016/j.cell.2016.03.046 PMC5503689 27153493

[25] Carpenter S, O’Neill LAJ (2024) From periphery to center stage: 50 years of advancements in innate immunity. Cell 187:2030–2051 10.1016/j.cell.2024.03.036 PMC11060700 38670064

[26] Boyd DF, Allen EK, Randolph AG, Guo XJ, Weng Y, Sanders CJ, Bajracharya R, Lee NK, Guy CS, Vogel P, Guan W, Li Y, Liu X, Novak T, Newhams MM, Fabrizio TP, Wohlgemuth N, Mourani PM, Investigators PPICI, Wight TN, Schultz-Cherry S, Cormier SA, Shaw-Saliba K, Pekosz A, Rothman RE, Chen KF, Yang Z, Webby RJ, Zhong N, Crawford JC, Thomas PG (2020) Exuberant fibroblast activity compromises lung function via ADAMTS4. Nature 587:466–471 10.1038/s41586-020-2877-5 PMC7883627 33116313

[27] Wang W, Li G, De W, Luo Z, Pan P, Tian M, Wang Y, Xiao F, Li A, Wu K, Liu X, Rao L, Liu F, Liu Y, Wu J (2018) Zika virus infection induces host inflammatory responses by facilitating NLRP3 inflammasome assembly and interleukin-1beta secretion. Nat Commun 9:106 10.1038/s41467-017-02645-3 PMC5760693 29317641

[28] Chen W, Foo SS, Zaid A, Teng TS, Herrero LJ, Wolf S, Tharmarajah K, Vu LD, van Vreden C, Taylor A, Freitas JR, Li RW, Woodruff TM, Gordon R, Ojcius DM, Nakaya HI, Kanneganti TD, O’Neill LAJ, Robertson AAB, King NJ, Suhrbier A, Cooper MA, Ng LFP, Mahalingam S (2017) Specific inhibition of NLRP3 in chikungunya disease reveals a role for inflammasomes in alphavirus-induced inflammation. Nat Microbiol 2:1435–1445 10.1038/s41564-017-0015-4 28848230

[29] Jiang T, Liu E, Li Z, Yan C, Zhang X, Guan J, Zhan Y, Zhao B, Ding W (2024) SIRT1-Rab7 axis attenuates NLRP3 and STING activation through late endosomal-dependent mitophagy during sepsis-induced acute lung injury. Int J Surg 110:2649–2668 10.1097/JS9.0000000000001215 PMC11093444 38445453

[30] Jiang YC, Zhao B, Jiang P, Wang SY, Ma L, Wu GL, Wang ML, Sun HM (2025) Raspberry ketone alleviates radiation-induced lung injury through the STAT2-P2X7r/NLRP3 signaling pathway. Phytomedicine 145:156984 10.1016/j.phymed.2025.156984 40544736

[31] Zhang X, Chen G, Yin J, Nie L, Li L, Du Q, Tong D, Huang Y (2024) Pseudorabies virus UL4 protein promotes the ASC-dependent inflammasome activation and pyroptosis to exacerbate inflammation. PLoS Pathog 20:e1012546 10.1371/journal.ppat.1012546 PMC11421794 39316625

[32] Munoz-Planillo R, Kuffa P, Martinez-Colon G, Smith BL, Rajendiran TM, Nunez G (2013) K(+) efflux is the common trigger of NLRP3 inflammasome activation by bacterial toxins and particulate matter. Immunity 38:1142–1153 10.1016/j.immuni.2013.05.016 PMC3730833 23809161

[33] Zhu J, Zhu R, Jiang H, Li Z, Jiang X, Li F, Zhang F, Feng X, Gu J, Li N, Lei L (2023) Adh promotes *Actinobacillus pleuropneumoniae* survival in porcine alveolar macrophages by inhibiting CHAC2-mediated respiratory burst and inflammatory cytokine expression. Cells 12:696 10.3390/cells12050696 PMC10001268 36899832

[34] Bahat A, Milenkovic D, Cors E, Barnett M, Niftullayev S, Katsalifis A, Schwill M, Kirschner P, MacVicar T, Giavalisco P, Jenninger L, Clausen AR, Paupe V, Prudent J, Larsson NG, Rogg M, Schell C, Muylaert I, Lekholm E, Nolte H, Falkenberg M, Langer T (2025) Ribonucleotide incorporation into mitochondrial DNA drives inflammation. Nature 647:726-734 10.1038/s41586-025-09541-7 PMC12629987 40993386

[35] Pomorska-Mol M, Dors A, Kwit K, Kowalczyk A, Stasiak E, Pejsak Z (2017) Kinetics of single and dual infection of pigs with swine influenza virus and *Actinobacillus pleuropneumoniae*. Vet Microbiol 201:113–120 10.1016/j.vetmic.2017.01.011 28284596

[36] Qi W, Zhu R, Bao C, Xiao J, Liu B, Sun M, Feng X, Gu J, Li Y, Lei L (2019) Porcine circovirus type 2 promotes *Actinobacillus pleuropneumoniae* survival during coinfection of porcine alveolar macrophages by inhibiting ROS production. Vet Microbiol 233:93–101 10.1016/j.vetmic.2019.04.028 31176418

[37] Wu C, Xu M, Dong J, Cui W, Yuan S (2024) The structure and function of olfactory receptors. Trends Pharmacol Sci 45:268–280 10.1016/j.tips.2024.01.004 38296675

[38] Zhu Z, Gong D, Chen Y, Yuan M, Qian H, He B, Wang G (2025) A review of research advances in the modulation of olfactory receptors for COPD inflammation and airway remodeling. Front Immunol 16:1612165 10.3389/fimmu.2025.1612165 PMC12270895 40688081

[39] Orecchioni M, Matsunami H, Ley K (2022) Olfactory receptors in macrophages and inflammation. Front Immunol 13:1029244 10.3389/fimmu.2022.1029244 PMC9606742 36311776

[40] Orecchioni M, Kobiyama K, Winkels H, Ghosheh Y, McArdle S, Mikulski Z, Kiosses WB, Fan Z, Wen L, Jung Y, Roy P, Ali AJ, Miyamoto Y, Mangan M, Makings J, Wang Z, Denn A, Vallejo J, Owens M, Durant CP, Braumann S, Mader N, Li L, Matsunami H, Eckmann L, Latz E, Wang Z, Hazen SL, Ley K (2022) Olfactory receptor 2 in vascular macrophages drives atherosclerosis by NLRP3-dependent IL-1 production. Science 375:214–221 10.1126/science.abg3067 PMC9744443 35025664

[41] Weidinger D, Jamal Jameel K, Alisch D, Jacobsen J, Burger P, Ruhe M, Yusuf F, Rohde S, Stortkuhl K, Kaufmann P, Kronsbein J, Peters M, Hatt H, Giannakis N, Knobloch J (2022) OR2AT4 and OR1A2 counterregulate molecular pathophysiological processes of steroid-resistant inflammatory lung diseases in human alveolar macrophages. Mol Med 28:150 10.1186/s10020-022-00572-8 PMC9743598 36503361

[42] Fan M, Zhang J, Tsai CW, Orlando BJ, Rodriguez M, Xu Y, Liao M, Tsai MF, Feng L (2020) Structure and mechanism of the mitochondrial Ca(^2+^) uniporter holocomplex. Nature 582:129–133 10.1038/s41586-020-2309-6 PMC7544431 32494073

[43] Busse D, Kudella P, Gruning NM, Gisselmann G, Stander S, Luger T, Jacobsen F, Steinstrasser L, Paus R, Gkogkolou P, Bohm M, Hatt H, Benecke H (2014) A synthetic sandalwood odorant induces wound-healing processes in human keratinocytes via the olfactory receptor OR2AT4. J Invest Dermatol 134:2823–2832 10.1038/jid.2014.273 24999593

[44] Kim JS, Lee HL, Jeong JH, Yoon YE, Lee IR, Kim JM, Wu C, Lee SJ (2022) OR2AT4, an ectopic olfactory receptor, suppresses oxidative stress-induced senescence in human keratinocytes. Antioxidants 11:2180 10.3390/antiox11112180 PMC9686838 36358552

[45] Xu X, Pang Y, Fan X (2025) Mitochondria in oxidative stress, inflammation and aging: from mechanisms to therapeutic advances. Signal Transduct Target Ther 10:190 10.1038/s41392-025-02253-4 PMC12159213 40500258

[46] Vringer E, Tait SWG (2023) Mitochondria and cell death-associated inflammation. Cell Death Differ 30:304–312 10.1038/s41418-022-01094-w PMC9950460 36447047

[47] Zhong Z, Liang S, Sanchez-Lopez E, He F, Shalapour S, Lin XJ, Wong J, Ding S, Seki E, Schnabl B, Hevener AL, Greenberg HB, Kisseleva T, Karin M (2018) New mitochondrial DNA synthesis enables NLRP3 inflammasome activation. Nature 560:198–203 10.1038/s41586-018-0372-z PMC6329306 30046112

[48] Fu J, Wu H (2023) Structural mechanisms of NLRP3 inflammasome assembly and activation. Annu Rev Immunol 41:301–316 10.1146/annurev-immunol-081022-021207 PMC10159982 36750315

[49] Qiu Y, Huang Y, Chen M, Yang Y, Li X, Zhang W (2022) Mitochondrial DNA in NLRP3 inflammasome activation. Int Immunopharmacol 108:108719 10.1016/j.intimp.2022.108719 35349960

