# Supplementary material for: Cooperation between Adh and OR5M11 contributes to the lung pathogenicity of Actinobacillus pleuropneumoniae by facilitating the NLRP3-mediated inflammatory response
Source: Vet Res. 2026 Oct 3;57:174. doi: 10.1186/s13567-026-01845-4 (PMC13633639; doi:10.1186/s13567-026-01845-4)
Supplement: Supplementary file 1 [file 13567_2026_1845_MOESM1_ESM.docx]

Additional file 1. The PCR primer pairs used in this study

| *Gene* | Species | **Sequence** |
| --- | --- | --- |
| *GAPDH* | Porcine | F:5’-GCCTGGAGAAACCTGCAAAAT-3’  R:5’-GACGAAGTGGTCGTTGAGGG-3’ |
| *OR5M11* | Porcine | F:5’-CTCGCTCTTGTTGCTACTCCT-3’  R:5’-GGCATTGGTGATGGGCACTT-3’ |
| *Caspase-1* | Porcine | F:5’-GCGTATTCAGAGCCGAGAGGGAG-3’  R:5’-CAGATTATGAGGGCAAGGCGTGT-3’ |
| *NLRP3* | Porcine | F:5’-GCCUUAAGUUGUGUGAAAUTT-3’  R:5’-UUCUCCGAACGUGUCACGUTT-3’ |
| *IL-1β* | Porcine | F:5’-ACGTGCAATGATGACTTTGTCTG-3’  R:5’-TGCAGAACACCACTTCTCTC-3’ |
| *TNF-α* | Porcine | F:5’-ACCACGCTCTTCTGCCTACT-3’  R:5’-CGGCTTTGACATTGGCTACAAC-3’ |
| *IL-6* | Porcine | F:5’-CTGGGTTCAATCAGGAGACCT-3’  R:5’-TTCCCTTTTGCCTCAGGGTC-3’ |
| *IL-18* | Porcine | F:5’-AGACCTGGAATCGGATTACTTTGGC-3’  R:5’-ACGGCTTGATGTCCCTGGTTAATG-3’ |
| *ASC* | Porcine | F:5’-AAAGCAGACAACAAACCAGCACT-3’  R:5’-CCGGAGTGAAGCTGAAGAGC-3’ |
| *MCP-1* | Porcine | F:5’-CTTGCCCAGCCAGATGCAAT-3’  R:5’-CCCACTTCTGCTTGGGTTCT-3’ |
| *cytb* | Porcine | F:5’-ATCCTGCCATTCATCATTACCG-3’  R:5’-TGATGAGATTCCGGTAGGGT-3’ |
| *COX1* | Porcine | F:5’-TCACCCGCAATACTATGAGCTCTG-3’  R:5’-GCCCCCTATAATGGCGAACAC-3’ |
| *D-loop* | Porcine | F:5’-CACACCCTATAACGCCTTGCC-3’  R:5’-GGGTAAGTGCCTGCTTTCGTAG-3’ |
| *NOX1* | Porcine | F:5’-CCGTAGCATTCCTCACCCTAGT-3’  R:5’-ATGGGTTGGAGTAGCCCGTAG-3’ |
| *NLRP3* | Mouse | F:5’-CCAGCCAGAGTGGAATGACA-3’  R:5’-GCGCGTTCCTGTCCTTGATA-3’ |
| *IL-1β* | Mouse | F:5’-AAATGCCACCTTTTGACAGTGATG-3’  R:5’-GCTCTTGTTGATGTGCTGCTG-3’ |
| *TNF-α* | Mouse | F:5’-ATGAGAAGTTCCCAAATGGC-3’  R:5’-CTCCACTTGGTGGTTTGCTA-3’ |
| *IL-6* | Mouse | F:5’-ATCGTGGAAATGAGAAAAGAGTTGT-3’  R:5’-ACTCCAGAAGACCAGAGGAAA-3’ |
| *IL-18* | Mouse | F:5’-CAACTTTGGCCGACTTCACTG-3’  R:5’-AGTCATATCCTCGAACACAGGC-3’ |
| *GAPDH* | Mouse | F:5’-TGTGTCCGTCGTGGATCTGA-3’  R:5’-TTGCTGTTGAAGTCGCAGGAG-3’ |
