# Supplementary material for: Cooperation between Adh and OR5M11 contributes to the lung pathogenicity of Actinobacillus pleuropneumoniae by facilitating the NLRP3-mediated inflammatory response
Source: Vet Res. 2026 Oct 3;57:174. doi: 10.1186/s13567-026-01845-4 (PMC13633639; doi:10.1186/s13567-026-01845-4)
Supplement: Supplementary file 2 [file 13567_2026_1845_MOESM2_ESM.docx]

1. (A-E) The relative quantification of indicated protein levels of Figure 2A; (F-K) The relative quantification of indicated protein levels.

2. (A and B) The relative quantification of indicated protein levels of Figure 4A; (C-E) The relative quantification of indicated protein levels Figure 4D; (F) The relative quantification of indicated protein levels Figure 4H; (G-H) The relative quantification of indicated protein levels Figure 4N.

3. (A) The relative quantification of indicated protein levels of Figure 5B; (C-G) The relative quantification of indicated protein levels Figure 5C; (H-J) The relative quantification of indicated protein levels Figure 5I.

4. (A) The relative quantification of indicated protein levels of Figure 6B; (B-E) The relative quantification of indicated protein levels Figure 6H.

5. The relative quantification of indicated protein levels of Figure 7F.
